# In Silico Prioritization of Variants of Uncertain Significance in ABCA4 Reveals Conserved Functional Motifs

**DOI:** 10.21203/rs.3.rs-10349982/v1

**Published:** 2026-08-25

**Authors:** Jazzlyn S. Jones, Barry Bodt, Subhasis B. Biswas, Esther E. Biswas-Fiss

**Affiliations:** University of Delaware; University of Delaware; University of Delaware; University of Delaware

**Keywords:** ABCA4, Stargardt disease, Extracytoplasmic domain 2 (ECD2), Variant effect predictor (VEP)

## Abstract

**Background:**

ABCA4 variants are the primary cause of Stargardt disease and also contribute to other inherited retinal disorders. Despite this central role, nearly half of all ABCA4 missense variants remain classified as variants of uncertain significance (VUS), limiting genetic diagnosis for many patients. The extracytoplasmic domain 2 (ECD2) harbors a disproportionate share of these unresolved variants yet remains poorly characterized.

**Methods:**

We analyzed 244 ECD2 missense variants using a consensus variant effect predictor (VEP) framework integrating PolyPhen-2, SIFT, and MutationTaster. Variants predicted pathogenic by all three tools were designated PAAT (pathogenic across all tools) and prioritized for further evaluation. PAAT variants were assessed using thermodynamic stability modeling ΔΔG and structural analysis, and alignment across ABCA family members was used to identify conserved regions, termed critical conserved motifs (CCMs).

**Results:**

PAAT variants were enriched in the central region of ECD2 (Pearson chi-square test, p = 0.0309) and showed a significantly higher frequency of destabilizing ΔΔG values than other variant categories (Cochran-Armitage trend test, p < 0.0001). PAAT variants had 2.2-fold higher odds of occurring within the four identified CCMs (95%: 1.29–3.81), directly linking predicted pathogenicity to sequence conservation. Structural modeling of select variants revealed specific disruptive mechanisms, including steric clashes and altered hydrogen bonding near the retinoid-binding interface.

**Conclusions:**

This integrative computational framework identifies four conserved motifs likely critical to ECD2 and ABCA4 function, and yields a prioritized, mechanistically grounded list of ECD2 variants for targeted experimental validation. These findings offer a practical path toward reclassifying ABCA4 VUS and improving genetic diagnosis for patients with Stargardt disease and related inherited retinal disorders.

## 1. Introduction

**Fig. 1:**
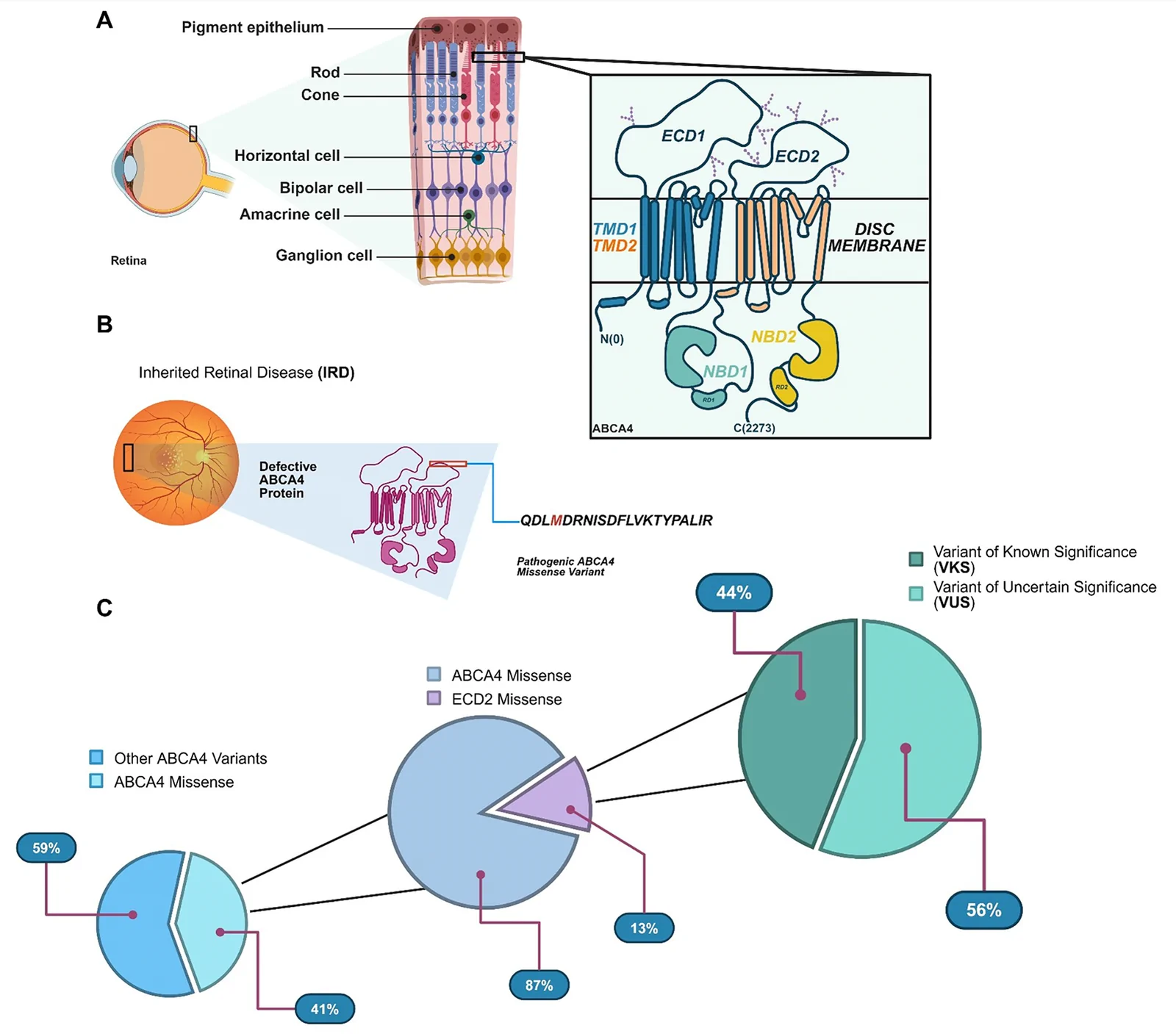
Subcellular Location of ABCA4 and Impact of ABCA4 Variants on Inherited Retinal Disease. (A) ABCA4 is a membrane protein that transports retinoid waste in the eye. It is located in the disc membrane (outer segment) of the rod and cone photoreceptor cells (B) Several inherited retinal diseases such as Stargardt disease (STGD1) are associated with pathogenic variants in the ABCA4 protein. Patient DNA sequencing has revealed many types of ABCA4 variants however missense variants represent the majority of variants found on the ABCA4 gene. (C) ABCA4 missense represent 41% of all ABCA4 variants while 59% represent other variant types such as frameshift, splice cite, and nonsense. ECD2 missense variants constitute a significant portion of ABCA4 missense variants (13%). The effect of many ECD2 variants on ABCA4 function is unknown and thus defined as variants of uncertain significance. ECD2 VUS are a large portion of ECD2 missense, totaling over 56%.Created in BioRender. Jones, J. (2026) https://BioRender.com/i3z8hb6.

ABCA4 (ATP Binding Cassette, Subfamily A, Member 4) and other members of the ABC protein family use energy from ATP hydrolysis to transport a variety of substrates across the cellular membrane. In humans, the ABCA4 protein is mainly expressed in the retina, specifically the rod and cone outer segments (Fig. 1A). The ABCA4 protein plays an essential role in vision where it is responsible for the transport and clearance of vitamin A derivatives known as retinoids. Over 4,600 ABCA4 variants have been identified, with many classified as missense, and deposited in databases such as ClinVar. Variants that disrupt ABCA4 function lead to toxic retinoid buildup, and ultimately to inherited retinal diseases (IRDs) including Stargardt disease(1–5), retinitis pigmentosa(3, 6–9), and cone-rod dystrophy(6–8, 10–13) (Fig. 1B). The process of retinoid transport is connected to disease which has allowed it to become a target in ABCA4-retinal dystrophy research. Understanding the process of retinoid transport is essential as it advances knowledge on ABCA4 related disorders.

ABCA4 is a member of the ABC transporter superfamily. This protein family, composed of 48 members divided into seven subfamilies (A to G), have considerable levels of sequence and structural homology (14, 15). The complete structure of an ABC transporter features a common core architecture with two transmembrane domains (TMDs) that bind substrates and two nucleotide-binding domains (NBDs) that hydrolyze ATP which are accompanied by cytoplasmic regulatory domains (RD) (16). The ABCA subfamily includes 12 full transporters and is distinguished from other ABC transporters by large extracytoplasmic domains (ECDs) located between the first and second transmembrane segments of each TMD (17).

Extracytoplasmic domain 2 (ECD2) extends from TMD2 of ABCA4 in a loop-like structure and has been shown to bind the retinoid all-*trans*-retinal (ATR), a byproduct of the visual transduction cycle (18). Recent cryo-EM studies have also reported that ECD2 forms a lid-tunnel-base structure with extracytoplasmic domain 1 (ECD1), implicating the domain in retinoid interaction and translocation (19–21). ECD2’s role in retinoid transport appears critical as it binds ATR and perhaps aids in its export out of the rod and cone outer segments, yet its overall role in the function of ABCA4 is still not fully understood.

The ECD2 domain, spanning ABCA4 residues 1391–1667, is 277 amino acids long and contains no known enzymatic or structural motifs. Of the 1,893 ABCA4 missense variants, nearly half are classified as variants of uncertain significance (VUS). 244 of these missense variants276 map to the ECD2 domain emphasizing the critical importance of the domain (22) (Fig. 1C). Characterization of these ECD2 variants is necessary, however due to the volume of these variants traditional biochemical assays are impractical. Instead, this research leverages the advanced *in silico* tools now available for protein analysis.

In this study, variant effect predictors (VEPs): Polyphen2, SIFT, and Mutation Taster are used to analyze ECD2 missense variants (23–25). VEPs use diverse algorithms to estimate variant pathogenicity and are increasingly applied in molecular genetics, particularly IRD research, where they typically supplement clinical findings (26). For each variant, predictions from the three tools were tallied and those with majority pathogenic scores were referred to as PAAT (pathogenic across all tools) and thus most likely to be disease-causing. PAAT variants were then examined to identify conserved regions, termed critical conserved motifs or CCMs. Variants classified as VUS, PAAT, or CCM, due to their potential functional impact, were assessed for local structural changes using PyMOL (27) and screened for predicted thermodynamic stability effects (ΔΔG) using DDMut (28). By applying various computational methods, we have identified potential ECD2 motifs that contribute to the function of both the domain and the ABCA4 protein.

## 2. Materials and Methods

### 2.1. Acquisition of ECD2 variants

ECD2 variants were retrieved from NIH’s database *ClinVar* by entering *ABCA4* in the general query field. ECD2 variants were selected from the list of ABCA4 variants using the amino acid location of the domain (1391–1667). The acquisition into the ECD2 domain resulted in a total of 244 variants.

### 2.2. Analysis of ECD2 variants

Each variant was entered into the following VEPs: Polyphen2, SIFT, Mutation Taster. Pathogenicity results for each variant from each database were gathered in tabular form. Variants were further classified according to the number of pathogenic scores received from the corresponding databases. For instance, zero pathogenic results classified a variant as pathogenic across zero tools (PAZT) while one pathogenic result classified a variant as pathogenic across one tool (PAOT), and two pathogenic results classified a variant as pathogenic across two tools (PATT).Variants receiving pathogenic predictions from all three tools were labeled as PAAT, indicating the greatest potential impact on protein function.

### 2.3. Phylogenetic analysis of ECD2 domain using Multiple Sequence Alignment (MSA)

ABCA family members were selected and aligned using the Clustal Omega MSA tool on the Jalview platform (29–31). Conserved scores were derived from the alignment and were scaled from 0–100 with 100 signifying the most conserved. These alignments with their corresponding conservation scores were visualized with the SnapGene 8.2.2 software (32). ECD2 critical conserved motifs (CCMs) were defined as sequence blocks that contained multiple moderately conserved residues, scores between 34–67, and highly conserved residues, scores between 67–100.

### 2.4. Observing local structural changes with PyMOL mutagenesis

ABCA4 cryo-EM structure (PDB ID: 7E7O), resolved by Xie et al. in 2021, was used as the template for analysis using PyMOL (version 3.1.6.1). Steric clashing and secondary structure changes such as hydrogen bond and salt bridge formation were observed using the mutagenesis wizard. Changes such as these were reported as significant if present in all rotamers of the variant amino acid.

### 2.5. Screening thermodynamic stability changes (ΔΔG) with DDMut

Thermodynamic stability changes for ECD2 variants were evaluated using the DDMut tool. The ABCA4 cryo-EM structure (PDB ID: 7E7O) was preprocessed by removing non-relevant ligands (retaining NRPE and PE) and repaired using YASARA software(33). The resulting ΔΔG values were then compared across all ECD2 variants.

### 2.6. Statistical evaluation of ECD2 variants

The Pearson chi-square test was used to evaluate the distribution of ECD2 variants by location. Levene’s test and the Cochran–Armitage trend test were applied to assess differences in ΔΔG values. For analyses involving ECD2 CCM regions, both a binomial test and the Cochran–Armitage test were used. All statistical analyses were performed using JMP software (version 19) (34).

## 3. Results and Discussion

### 3.1. Comparative statistics of variant effect predictors

**Fig. 2:**
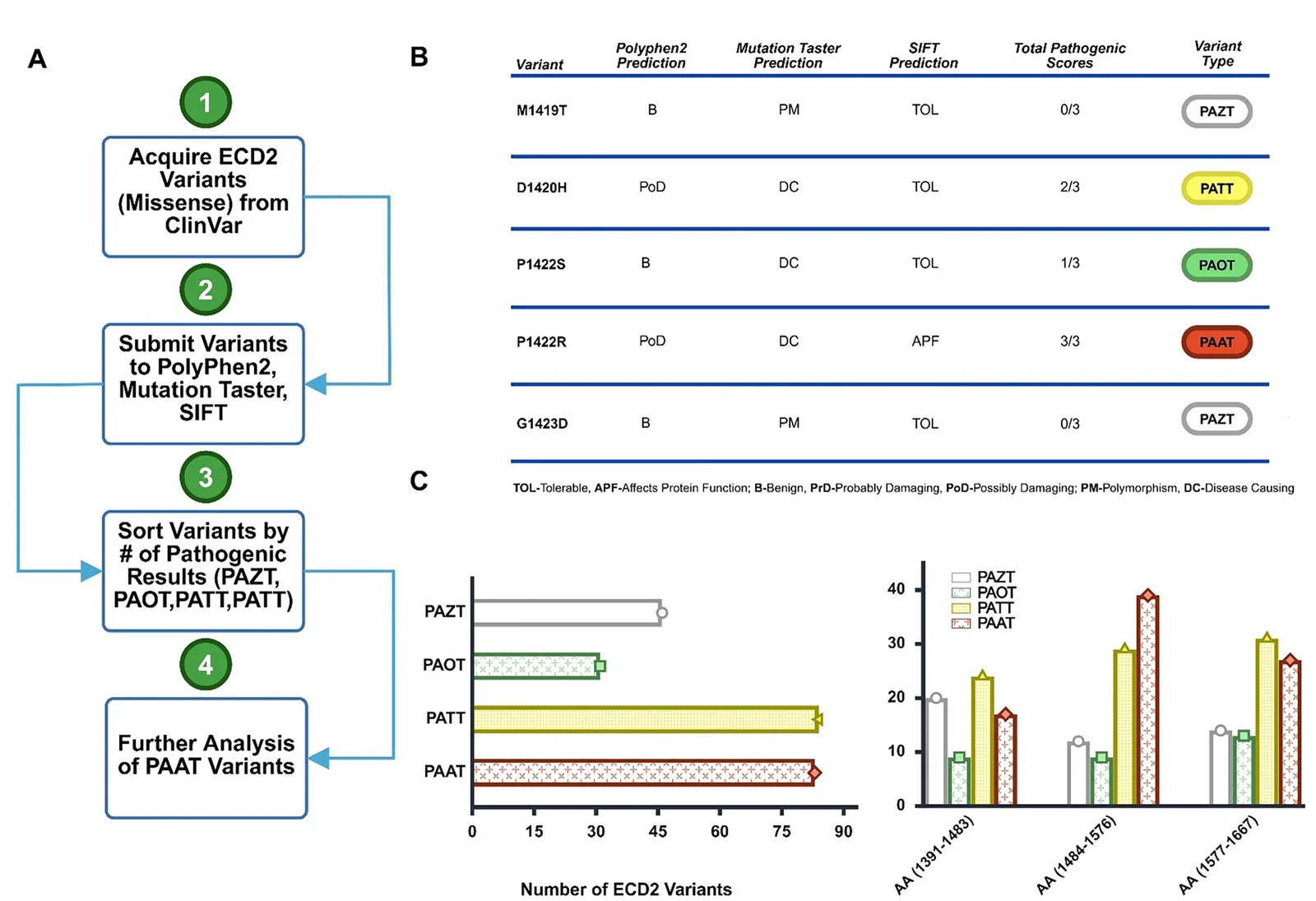
*In* silico prediction and assessment of ECD2 missense variants. (A) ECD2 variants were acquired from NIH database ClinVar and analyzed by the following *in silico* predictors: PolyPhen2, Mutation Taster, and SIFT. Each variant was further classified by the total number of pathogenic scores received. Variants with 0 pathogenic scores were labeled PAZT (pathogenic across zero tools), 1 pathogenic score were labeled as PAOT, 2 pathogenic scores were labeled as PATT, and 3 pathogenic scores were labeled as PAAT (B) Predictions from *in silica* tools and variant type (PAZT, PAOT, PATT, PAAT) are shown for five N-terminal ECD2 variants. (C)The total number (left) and distribution (right) of PAZT, PAOT, PATT, and PAAT variants is shown. Created in BioRender. Jones, J. (2026) https://BioRender.com/bbmm0s2.

The ECD2 variants (244 total) were evaluated using Polyphen2, MutationTaster, and SIFT (Fig. 2A). Of these, 46 variants (18.9%) received a PAZT classification, indicating pathogenic predictions from zero tools. The PAOT category, representing pathogenic predictions from one tool, included 31 variants (12.7%). The PATT and PAAT categories, pathogenic across two tools and across all three tools, contained 84 (34.4%) and 83 (34.0%) variants, respectively (Fig. 2B and 2C). Our VEP based classification system more frequently identified variants in the more detrimental categories (PATT and PAAT) than those in the benign categories (PAZT and PAOT). The tendency to classify variants as pathogenic is expected as it is observed in the individual VEP assessments.

ECD2 variants were considered pathogenic by Mutation Taster 76.2% of the time compared to 67.6% with Polyphen2, and 39.8% with SIFT.

PAZT, PAOT, PATT, and PAAT variants were also quantified by region of the ECD2 domain. ECD2 amino acids 1391–1483 were considered the start of the domain, amino acids 1484–1576 were the mid region, while amino acids 1577–1667 were the terminal region. Most PAAT variants were located in the mid region, with 39 identified (47%) (Fig. 2C).

The distributions of PAAT verses non PAAT for variants over the three ECD2 regions were tested for equivalence using a 2 × 3 contingency table. This analysis indicated that PAAT and non PAAT variants are not distributed in the same way among the regions, as supported by the Pearson chi-square test (N = 244, X^2^_2_=7.0, p = 0.0309). The adjusted Pearson residual for PAAT variants in the mid ECD2 region was 2.45 compared with − 2.03 in the start region. These residuals indicate an overrepresentation of PAAT variants in the mid ECD2 region relative to expectation.

The most adverse category of variants, PAAT, being found primarily in the mid region suggests the functional importance of this location to the ECD2 domain. Conversely, there were 20 PAZT variants at the start of the domain, representing the most prominent VEP class in this region. The presence of PAZT, which are most likely to be benign, at the start of ECD2 signifies this region may have less contributions to the functional activity of the domain. Overall, examining how these categories map across ECD2 has enabled the identification of potential functional hotspots on the domain.

Regarding accuracy, the PAAT category was able to capture 38 out of 69 (55.1%) pathogenic variants, as identified in NIH’s ClinVar database. When PATT and PAAT categories were combined, 64 pathogenic variants were identified, increasing the percentage captured to 92.8%. The ability of our VEP system to identify pathogenic variants aligns with individual tool capabilities. Polyphen 2 identified 65 (94.2%) pathogenic variants, followed by MutationTaster identifying 67 (97.1%) and SIFT identifying 38 (55.1%).

### 3.2. Estimated thermodynamic stability changes (ΔΔG) of ECD2 variants

**Fig. 3:**
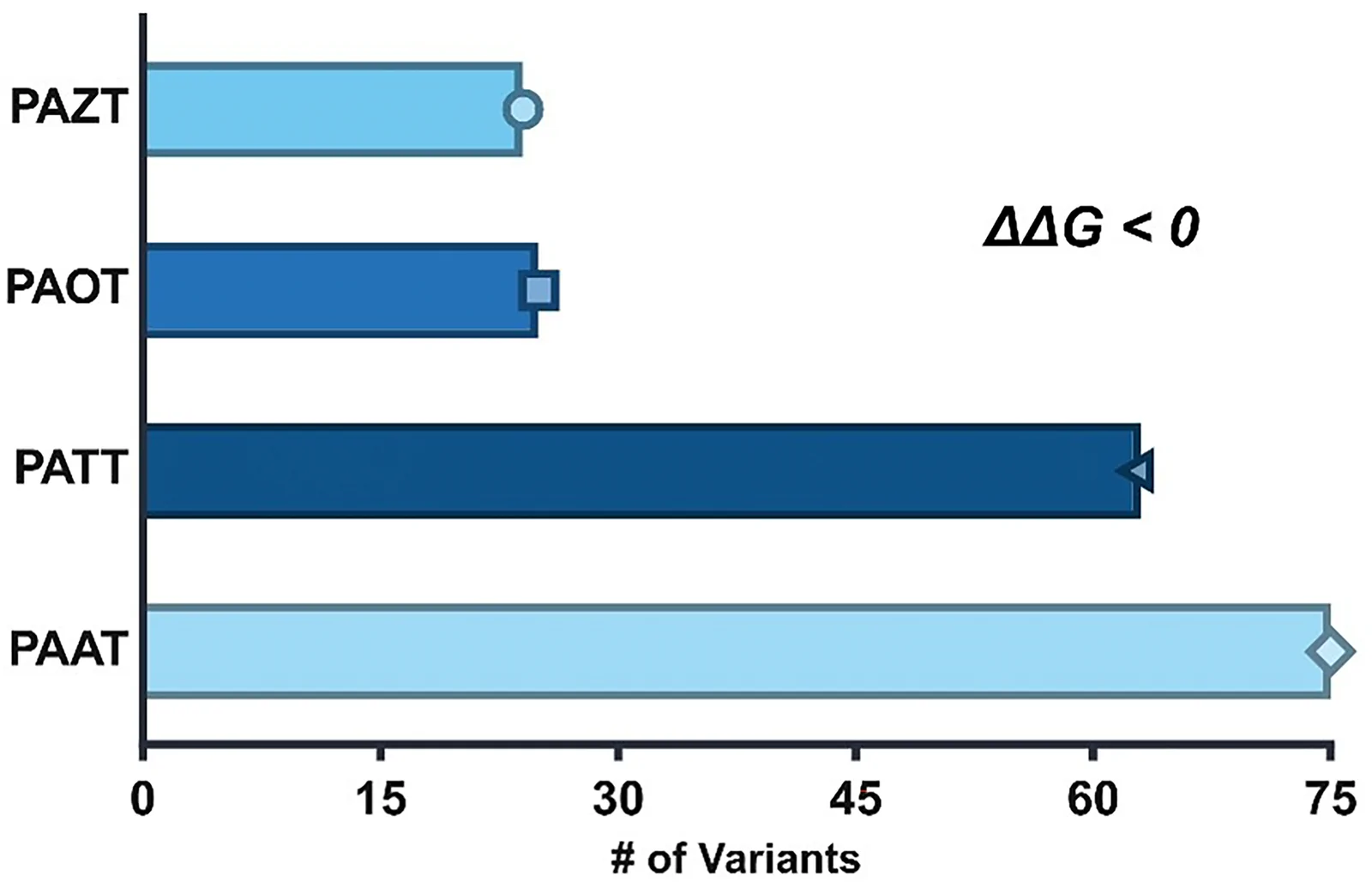
Predicted Gibbs Free Energy Changes (ΔΔG) of ECD2 Variants.Estimations of Gibbs free energy changes for each ECD2 variant were generated using DDMut. Variant specific ΔΔG values were compared to the ABCA4 wild type reference to identify mutations associated with destabilizing energetic shifts. ΔΔG distributions were further analyzed across the four ECD2 variant categories (PAZT, PAOT, PATT, and PAAT). Created in BioRender. Jones, J. (2026) https://BioRender.com/J35ddi8.

Estimation of ΔΔG values across PAZT, PAOT, PATT, and PAAT categories was performed using DDMut with stabilizing values reported as > 0 kcal/mol and destabilizing as < 0 kcal/mol (Fig. 3).

Means, standard deviations, 10th, and 90th percentile are given for ΔΔG values for VEP classes PAZT (−0.18, 0.36, −0.67, 0.11), PAOT (−0.38, 0.59, −1.10, 0.04), PATT (−0.67, 0.78, −1.85, 0.08), and PAAT (−1.34, 1.10, −2.88, −0.01). Levene’s test rejected equal variances across VEP classes (F[3, 240] = 21.8, p < 0.0001) with standard deviations seen to increase with increasing VEP tests. Welch’s ANOVA rejected the hypothesis of equal means (F[3, 108] = 27.5, p < 0.0001) with means generally decreasing with increasing VEP tests.

Regarding the quantity of destabilizing values within each VEP class, the PAZT category presented the least number of destabilizing variants with a total of 24 out of 46 (52.2%). PAOT had a total of 25 out of 31 (80.6%) while PATT had a total of 63 out of 84 (75.0%) variants. PAAT had the highest percentage of destabilizing variants with a total of 75 out of 83 (90.4%). A Cochran-Armitage test, using a 2 × 4 contingency table with destabilizing/stabilizing ΔΔG by pathogenic predictive ordering of VEP (PAZT, PAOT, PATT, PAAT), showed a significant increasing trend in the proportion of destabilizing ΔΔG over VEP (Z = 4.52, p < 0.0001). The presence of potential destabilizing effects among the likely pathogenic ECD2 variants such as PAAT resembles what is typically observed experimentally for known pathogenic variants (35). Detecting similar patterns across ECD2 variants further supports the validity of our VEP system.

### 3.3. Organization and diversity of ECD2 CCM regions

**Fig. 4:**
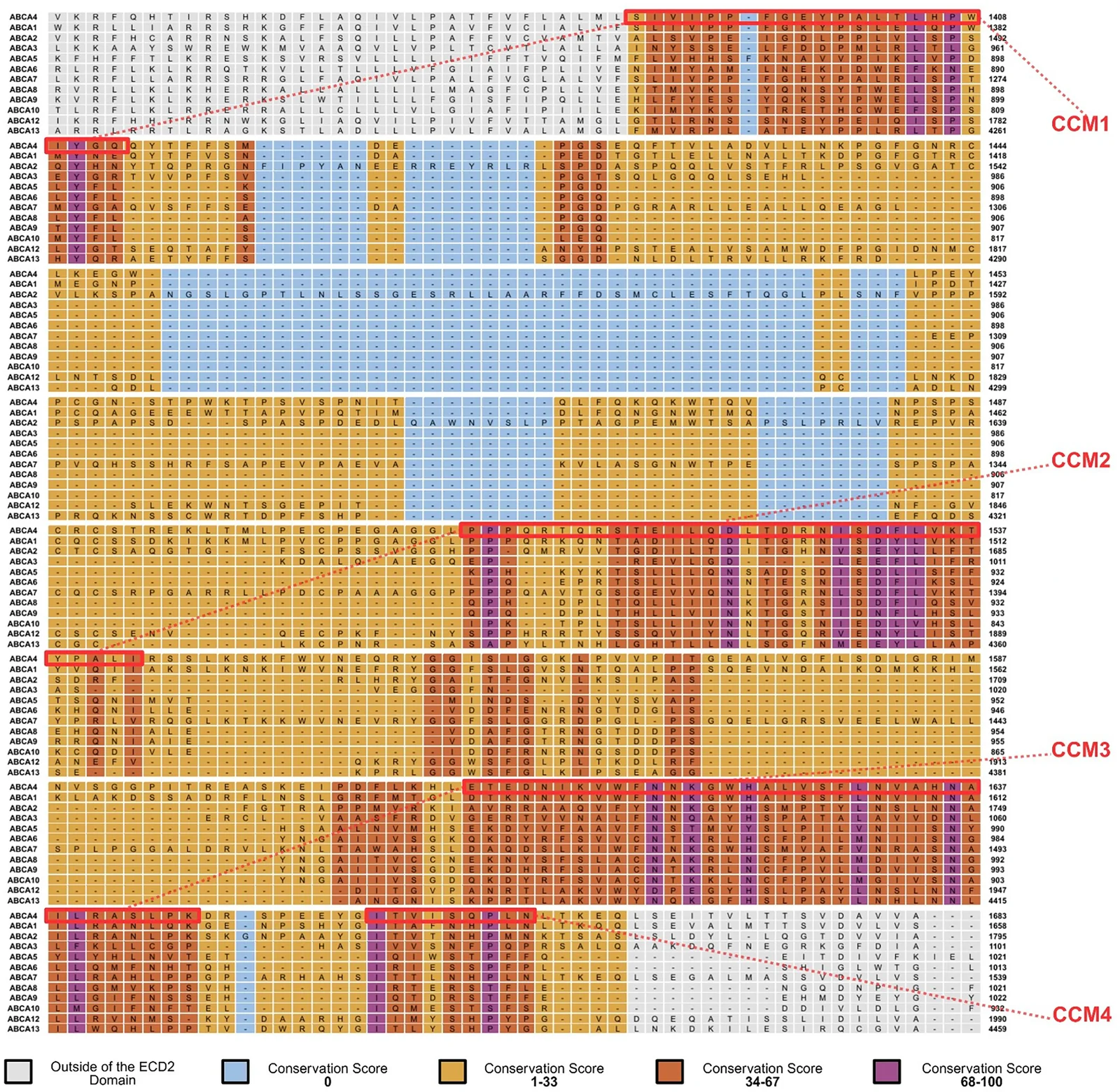
Multiple sequence alignment (MSA) of the ECD2 domain. Sequences from protein members of the ABCA subfamily were aligned using Clustal Omega multiple sequence alignment. Conservation of each amino acid in the MSA was calculated using the Jalview platform which employs the AMAS (analysis of multiply aligned sequences) method. Grey regions of the MSA highlight regions outside of the ECD2 domain and were therefore not included in the conservation analysis. Blue regions indicated a conservation of 0, light orange indicated a conservation from 1–33, dark orange indicated a conservation from 34–67, while purple indicated the highest conservation with a score from 68–100. MSA of the ECD2 domain revealed 4 conserved regions defined as critical conserved motifs (CCMs). CCM locations are as follows: CCM1:1392–1412, CCM2: 1510–1542, CCM3:1610–1645, CCM4: 1654–1662. Each CCM is highlighted in red. Created in BioRender. Jones, J.(2026)https://BioRender.com/o16fcok.

ECD2 sequences amongst the ABCA subfamily members were aligned (Fig. 4). Isolating blocks of conserved amino acids from the alignment revealed distinct conserved regions, termed critical conserved motifs (CCMs). The first of these regions, designated CCM1, spans ECD2 amino acids 1392–1412; CCM2 spans 1510–1542; CCM3 spans 1610–1645; and CCM4 spans 1654–1662.

To better understand the structural context of these motifs, the CCM regions were visualized on the ABCA4 cryo-EM structure (PDB ID: 7E7O) (Supplementary Material 2)(Fig. S1A). Although the CCMs contain all major forms of secondary structure, they are predominantly coiled, with 47.5% of residues adopting a coil conformation (Supplementary Material 2)(Fig. S1B). This high degree of flexibility is consistent with the dynamic nature expected for the ECD2 domain.

**Fig. 5:**
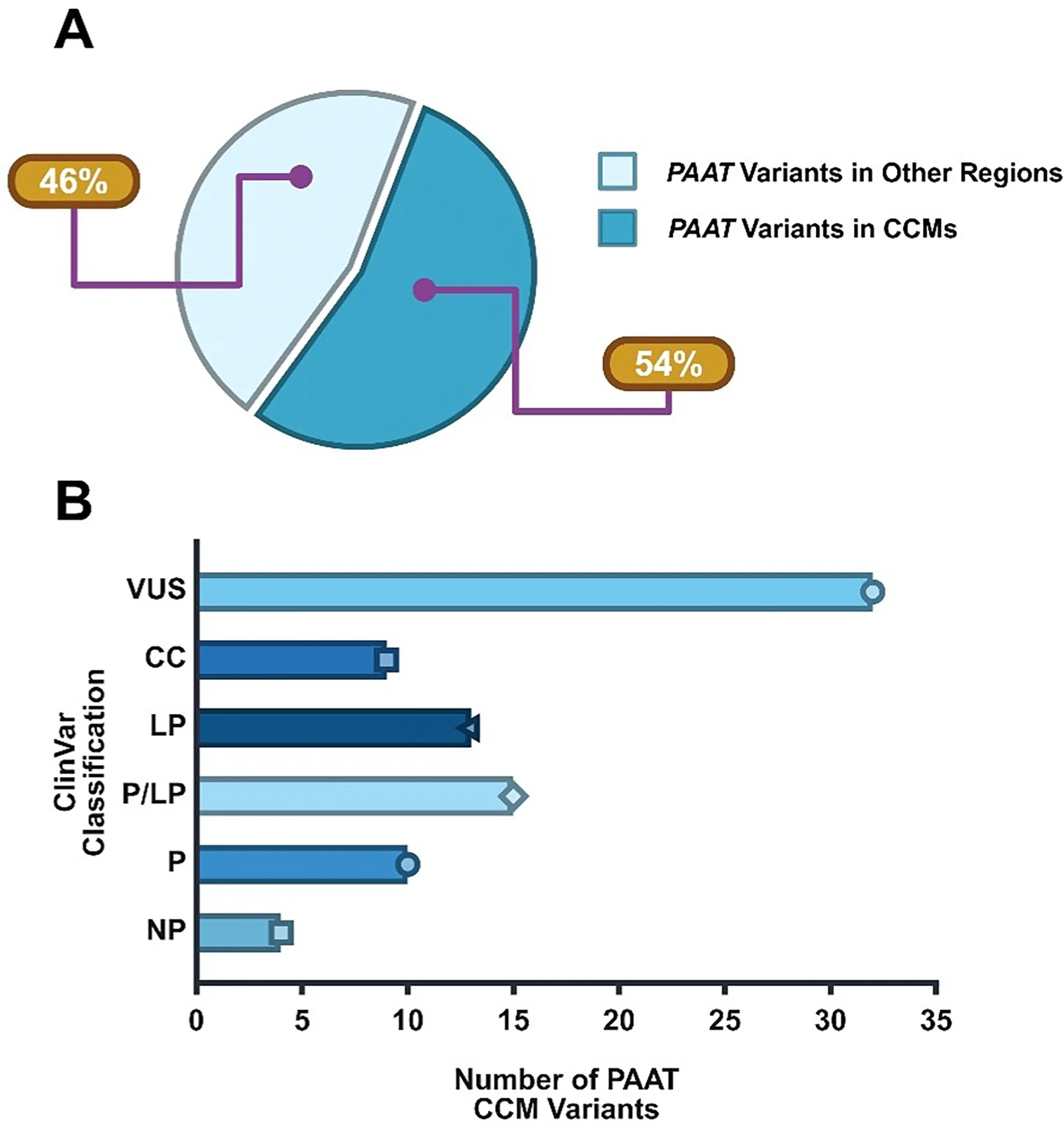
Intrinsic features of ECD2 PAAT variants. (A) The number of PAAT variants within CCM regions was compared to PAAT variants in other regions. (B) PAAT CCM variants were quantified according to their classification in NIH’s ClinVar database. ClinVar classifications are as follows VUS= variants of uncertain significance, CC= conflicting classifications, LP=likely pathogenic, P/LP= pathogenic/likely pathogenic, P=pathogenic, NP= not provided.Created in BioRender. Jones, J. (2026) https://BioRender.com/5dis478.

Following the designation of the CCM regions and assessing their structural context, the distribution of VEP class variants (PAZT, PAOT, PATT, PAAT) across these motifs was determined. A Cochran-Armitage test, using a 2 × 4 contingency table with CCM or non CCM regions by pathogenic predictive ordering of VEP (PAZT, PAOT, PATT, PAAT), showed a significant increasing trend in the proportion of CCM regions over VEP ($Z = 3.63, p < 0.0001$). To focus on PAAT, the table was collapsed to a 2 × 2 contingency table with PAAT and non PAAT VEP classes by CCM and non CCM regions. A disproportionate and significant number of PAAT variants (45, 54.2%) was found in the CCM regions (N = 244, Chi-square (1) = 8.5, p = 0.0035) with the odds of a PAAT prediction in a CCM region 2.22 times the odds of a non PAAT prediction in a CCM region, 95% CI (1.29, 3.81)(Fig. 5A). The analysis indicates that the CCMs are enriched for variants predicted to affect ECD2 function such as those in the PAAT VEP class. This finding implicates the potential importance of CCMs in the structural and functional integrity of the ECD2 domain as well as the ABCA4 protein. The ClinVar profiles of PAAT CCM variants were also examined which revealed most variants were classified as VUS followed by pathogenic designations (LP, likely pathogenic; P/LP, pathogenic/likely pathogenic; or P, pathogenic) (Fig. 5B).

**Fig. 6:**
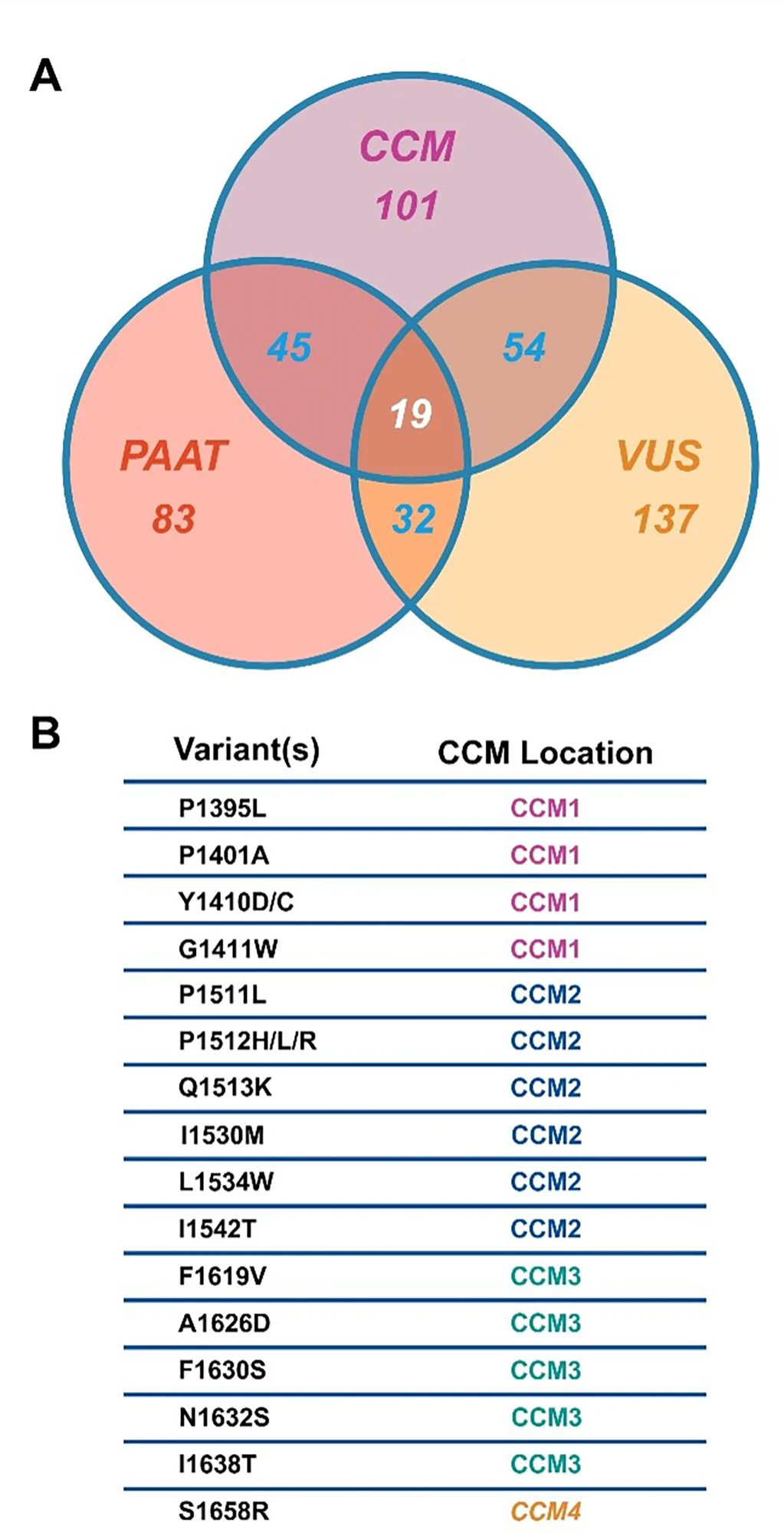
Overlap and Distribution of ECD2 PAAT, VUS, and CCM Variant Classes. (A) Venn Diagram indicates overlap between ECD2 variant categories including PAAT, VUS, and CCM. Each category was quantified with PAAT represented in red, VUS in orange, and CCM in magenta. The number of variants amongst all categories is represented in white. (B) ECD2 PAATNUS/CCM variants are listed with their corresponding CCM. Multiple variants at the same amino acid location are indicated by the forward slash.Created in BioRender. Jones, J. (2026) https://BioRender.com/58tjloj.

Among CCM regions with PAAT prediction, an exact binomial test found that CCM2 and CCM3 had a significantly higher number of PAAT variants (34, p = 0.0008) than combined CCM1 and CCM4. Physical mapping of these variants showed that CCM2 and CCM3 contained the highest number of PAAT variants (18) while also emphasizing the many PAAT variants which are classified as VUS (Supplementary Material 2)(Fig.S2). The ECD2 PAAT/VUS/CCM subgroup provides potential insight into the mechanisms of the domain and presents clinical implications for VUS reclassification (Fig. 6). For these reasons, they were the focus of the subsequent in-depth structural evaluation of the ECD2 domain.

### 3.4. Structural assessment of PAAT and VUS variants in ECD2 CCM regions

**Fig. 7:**
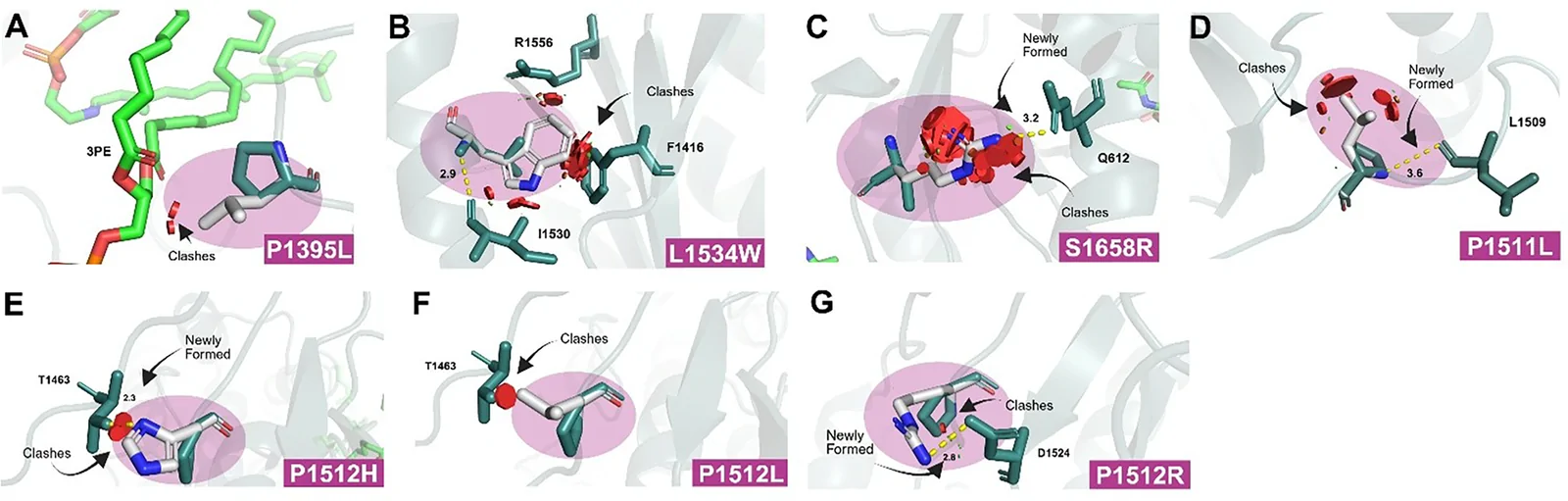
Structural assessment of PAAT and VUS variants in ECD2 CCM regions. ECD2 variants classified as PAAT, VUS, and also present in CCM regions were visualized in PyMOL.Wild type residues are shown in teal with corresponding variant residues in gray (see purple oval). Surrounding residues are labeled. Regions with steric clashing are represented as red discs while hydrogen bond formation is indicated in yellow with distance (angstrom) labeled. (A) P1395L exhibits steric clash near the 3PE substrate. (B) L 1534W shows clashing among neighboring ECD2 residues. (C) S1658R displays extensive steric clashing and forms a new hydrogen bond with ECD1 residue 0612. (D) P1511L variant presents steric clashing and new hydrogen bond with ECD2 residue L 1509.(E·G) All variants at the P1 512 locus demonstrate clashing with nearby residues. P1512H and P1 512R additionally form new hydrogen bonds with surrounding ECD2 residues. Created in BioRender. Jones, J. (2026) https://BioRender.com/z56efnm.

Majority of the ECD2 PAAT/VUS CCM subgroup demonstrated various dynamic local structure changes. The P1395L variant showed minor steric clashing at the retinoid binding site specifically with the 3PE (3-sn-phosphatidylethanolamine) substrate (Fig. 7A). Steric hindrance in this location could have notable consequences as 3PE has been shown to stabilize ABCA4 and improve NRPE (N-retinylidene-phosphatidylethanolamine)-stimulated ATPase activity (36, 37). Additional variants displayed steric clashing including L1534W, S1658R, P1511L and all variants at the P1512 locus (Fig. 7B–G). S1658R, P1512H, and P1512R variants also formed new hydrogen bonds with other ABCA4 residues. Specifically, S1658R established a new interaction with ECD1 residue Q612, while P1511L, P1512H, and P1512R formed new bonds with ECD2 residues L1509, T1463, and D1524 respectively. A1626D introduced steric clashing near intradomain residues Y1410 and Y1557 (Supplementary Material 2)(Fig. S3A). The I1638T variant was present on an alpha helix and incorporated a new hydrogen bond within the ABCA4 structure (Supplementary Material 2)(Fig.S3B). Modifications such as these may cause restrictions in the alpha helices which may impact the intrinsic flexibility of the protein. There were also some ECD2 variants, F1619V and N1632S, that displayed contrast behavior with the wild type amino acids having moderate steric clashing while variant amino acids displayed minimal or zero overlap (Supplementary Material 2) (Fig. S3C-F).

## 4. Conclusion

With our custom VEP system we have achieved a manageable workflow which can identify potentially pathogenic variants, PAAT, in the ECD2 domain. Our method is high throughput when considering an immense number of variants and is a suitable approach as it is comparable to standard variant prediction methods.

ECD2 PAAT variants emerged as a distinct class of ECD2 variants, with many exhibiting characteristics that are suggestive of pathogenic variants. These types of variants can implement drastic structural changes which can be demonstrated by changes in Gibbs free energy (38). PAAT variants exhibited these same behaviors with the majority (90.4%) possessing destabilizing ΔΔG values.

Many variants highlighted in the structural modeling of the domain also displayed substantial structural impacts on the domain. ECD2 variant P1395L displayed steric clashing near one PE (3PE) molecule within the retinoid binding site. A variant in this location can be potentially damaging as it has been reported that interaction with PE increases ATPase activity of the ABCA4 protein. This variant lies at the same site where a conformational change is observed in the retinoid-bound ABCA4 cryo-EM structure (PDB ID:7E7O), suggesting that the P1395 position may play an important role in the domain’s structural and functional organization.

L1534W, A1626D, S1658R, and P1512 variants all demonstrated steric clashing while the I1638T variant displayed changes in the alpha helix structure. Changes induced by the new hydrogen bonds can alter the overall flexibility of the domain, an aspect that can affect the overall stability of the ECD2 domain and the ABCA4 protein.

Variants P1512H and P1512R each formed new hydrogen bonds with surrounding ECD2 residues. These additional interactions have the potential to alter the local environment including the secondary structure of the ECD2 domain. All variants at the P1512 locus demonstrated localized structural changes supporting the idea that this position may be critical for ECD2 function and identifying this position as a promising site for future biochemical studies. The S1658R variant also formed a new hydrogen bond. Notably this bond was formed with the ECD1 residue Q612. ECD1 and ECD2 together form the dynamic tunnel that guides retinoid transport. Therefore, this new interdomain interaction in S1658R may influence how the two domains coordinate and ultimately how they contribute to ABCA4 function.

Examination of the PAAT group not only revealed local structural changes but also potential functional hotspots or patterns within ECD2. Consistent with this, 47% of PAAT variants clustered within ECD2’s mid region residues 1484–1576. Prior biochemical studies have demonstrated ECD2’s specificity for the retinoid ATR and its role in retinoid transport. The enrichment of variants in this region suggests that residues 1484–1576 may be critical for ECD2 function and potentially involved in retinoid interaction.

Together, in silico prioritization followed by targeted functional characterization offers a high-throughput strategy for evaluating ABCA4 variants. Streamlining functional assessment particularly for ECD2 VUS will support more accurate variant interpretation and classification. As the number of disease-associated variants in ECD2 and across ABCA4 continues to grow, reliable and efficient approaches such as these will be essential. Ultimately, detailed ECD2 variant studies will strengthen our understanding of the domain’s contribution to ABCA4 function and broaden insights into ABCA4-associated inherited retinal diseases.

## Supplementary Material

This is a list of supplementary files associated with this preprint. Click to download.

• JJonesSupplementaryMaterial1.xlsx

• JJonesSupplementaryMaterial2.docx

• JJonesGraphicalAbstract.jpeg

## Data Availability

The ECD2 variant dataset generated in this study is provided as a supplementary material file (Supplementary Material 1).

## Materials availability

Not applicable to this study.

## Code availability

Not applicable to this study.

## Abbreviations

ABCA4: ATP-binding cassette sub-family A member 4
ECD2: Extracytoplasmic domain 2
VUS: Variant of uncertain significance
VEP: Variant effect predictor
PAAT: Pathogenic across all tools
CCM: Critical conserved motif
IRD: Inherited retinal disease
TMD: Transmembrane domain
NBD: Nucleotide-binding domain
RD: Regulatory domain
ATR: all-trans-retinal
ECD1: Extracytoplasmic domain 1
PAZT: Pathogenic across zero tools
PAOT: Pathogenic across one tool
PATT: Pathogenic across two tools
LP: Likely pathogenic
P/LP: Pathogenic/likely pathogenic
P: Pathogenic
3PE: 3-sn-phosphatidylethanolamine
NRPE: N-retinylidene-phosphatidylethanolamine
MSA: Multiple sequence alignment
